# Multispectral UAV imaging and machine learning for estimating wheat nitrogen nutrition index

**DOI:** 10.3389/fpls.2025.1709459

**Published:** 2025-12-15

**Authors:** Chao Zhang, Xinyi Lu, Haolei Zhang, Hongwei Cui, Hao Ma, Jiangtao Ji, Tianhang Ding

**Affiliations:** 1 Key Laboratory of Modern Agricultural Equipment, Ministry of Agriculture and Rural Affairs, Nanjing, China; 2 College of Agricultural Equipment Engineering, Henan University of Science and Technology, Luoyang, China; 3 Zhejiang Key Laboratory of Intelligent Sensing and Robotics for Agriculture, College of Biosystems Engineering and Food Science, Zhejiang University, Hangzhou, China

**Keywords:** multispectral, nitrogen application level, nitrogen nutrition index, planting density, spring wheat, vegetation indices

## Abstract

**Introduction:**

Monitoring nitrogen nutrition indices is crucial for assessing current wheat growth conditions and guiding nitrogen fertilizer application.

**Methods:**

To estimate the wheat nitrogen nutrition index (NNI) and explore the effects of planting density and nitrogen application rates on NNI, this study employed UAVs to capture multispectral canopy imagery of wheat at key growth stages (tillering, jointing, booting, and filling) under varying planting densities and nitrogen application rates. Vegetation indices were selected using Pearson correlation and feature importance analysis. A Bayesian optimized random forest model was constructed to estimate the NNI.

**Results:**

Experimental results indicate that vegetation indices DVI, MDD, NGI, MEVI, NDVI, EVI, and ENDVI exhibit strong resistance to interference, enabling the construction of highly robust models. The NNI estimation model developed under nitrogen application level N2 (210 kg/hm^2^) demonstrated optimal performance, with R^2^ and RMSE values of 0.785 and 0.137, respectively. The NNI estimation model constructed at planting density P1 (1 million plants/hm^2^) was optimal, with R^2^ and RMSE of 0.716 and 0.158, respectively. It was also found that NNI generally exhibited an initial increase followed by a decrease as planting density increased.

**Discussion:**

The research findings systematically reveal the patterns of planting density and nitrogen application levels affecting wheat NNI. The constructed NNI estimation model plays a crucial role in assessing wheat growth status and also provides reference for rationally determining planting density and nitrogen application levels for spring wheat.

## 1 Introduction

Spring wheat is a globally important staple crop whose production underpins food security and agricultural economic stability. Nitrogen is essential for spring-wheat growth, with supply levels directly affecting plant performance, yield, and grain quality. The Nitrogen Nutrition Index (NNI) serves as a critical indicator of crop N status and is widely employed to quantify nitrogen sufficiency, guiding precision fertilization (Li et al., 2024). Reliable NNI estimation enhances nitrogen-use efficiency, curbs fertilizer losses, reduces environmental impacts, and supports sustainable farming. Thus, real-time, rapid, and non-destructive NNI estimation carries both theoretical and practical significance.

Planting density (P) and nitrogen application level (N) rate are key drivers of crop performance. Density dictates plant population and spatial arrangement, thereby modulating competition and resource-use efficiency, while N rate governs soil-N availability and plant N status. The coupled effect of planting density and nitrogen application level alters soil physicochemical properties, bacterial community structure and function, and root physiology, ultimately shaping nitrogen-use efficiency (Liang et al., 2024). At low densities, additional N markedly increases both the efficiency and relative contribution of N translocation to grain filling; at high densities, these gains are negligible (Ren et al., 2017). This indicates that the interplay between planting density and N rate imposes a complex control over spring-wheat N nutrition. Clarifying this interaction is therefore essential for refining management practices and enhancing fertilizer-use efficiency in spring-wheat systems.

Current remote sensing approaches for estimating wheat NNI primarily encompass satellite remote sensing, hyperspectral instrumentation, and multispectral instrumentation (Zhao et al., 2014). Satellite imagery is widely utilized for large-scale crop growth monitoring. However, sensors like SPOT, MSS, and Landsat feature low spatial resolution, which falls short for precise nitrogen nutrition diagnosis at the plant scale. Although hyperspectral instruments have high resolution, the data collected is redundant, and screening effective data is cumbersome (Tian et al., 2013). Amid the swift advancement of crop science research, UAV-based multispectral proximal remote sensing has emerged as a robust and practical solution (Cao et al., 2012). It surmounts the limitations of low resolution and poor timeliness in satellite remote sensing, as well as the data redundancy and operational intricacies of hyperspectral instruments. Boasting high spatial resolution, flexible temporal resolution, convenient operation, and efficient multispectral data collection (Azizabadi et al., 2025), this technology enables fine-scale nitrogen nutrition diagnosis of crops at both field and individual plant levels. To quickly and accurately estimate the NNI during the early panicle differentiation stage of rice, Li et al (Li et al., 2025). conducted a three-year field experiment with five nitrogen levels and ten rice cultivars, who collected NNI and UAV multispectral data during this stage and constructed NNI estimation models by combining three feature selection methods with four machine learning algorithms. The model using vegetation indices as inputs achieved an R^2^ of 0.8767, RMSE of 0.0549, and MAE of 0.0383. Liu et al (Liu et al., 2018). collected NNI and UAV multispectral data during four critical growth stages of winter wheat and developed NNI estimation models for each stage and the entire growth period. The R^2^ for these models ranged from 0.45 to 0.96. Pei et al (Pei et al., 2023). collected NNI and UAV multispectral data throughout the entire growth period of cotton canopies and developed three machine learning models for NNI estimation using vegetation indices as inputs. Among these, the XGB model performed the best, achieving an R^2^ of 0.65 and an RMSE of 0.09. Although the above studies have made progress in estimating NNI using UAV multispectral technology combined with machine learning algorithms, most of them have focused on the model construction itself. These studies are useful for assessing nitrogen utilization efficiency in wheat, but they have not systematically investigated the pattern of planting agronomy (e.g., planting density, nitrogen application level, etc.) on NNI estimation. This limitation leads to the difficulty of existing models to provide targeted references for optimizing planting management in actual production.

Aiming at the problem that current research focuses on modeling but neglects the lack of understanding of planting agronomic regulatory mechanisms, this study designed a three-factor, three-level, full-factor field experiment using variety, planting density, and nitrogen application level as the influencing factors, and multispectral image data of spring wheat at different fertility stages were collected. Pearson’s correlation and character importance were used to dynamically screen vegetation indices. Bayesian optimized random forest (BO-RF) algorithm was introduced to construct the NNI estimation model. The aim of this study was to systematically investigate the influence patterns of planting density and nitrogen application level on NNI estimation in spring wheat and construct NNI estimation models under different planting conditions. This study has the following research objectives: (1) to determine the optimal NNI estimation model for spring wheat under different planting densities and nitrogen application levels; (2) to analyze the influence of the coupling effect of P and N on the estimation of NNI in spring wheat; and (3) to screen the highly robust vegetation indices applicable to each treatment.

## 2 Materials and methods

### 2.1 Technical route

**Figure 1:**
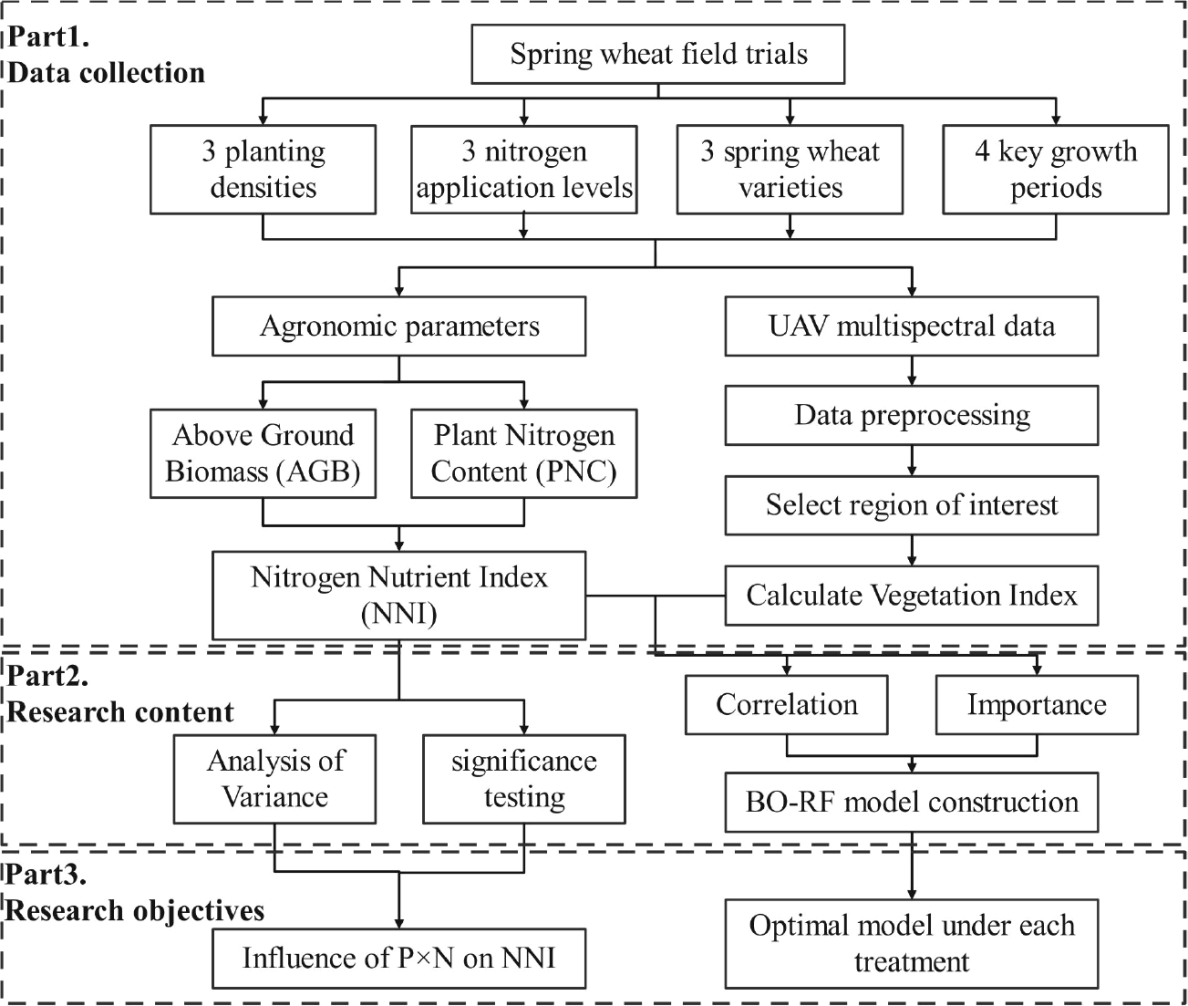
Technical route.

The technical route of this study is illustrated in **Figure 1**. First, UAV multispectral remote sensing data and agronomic parameters of spring wheat were collected. The multispectral canopy data were then preprocessed, including radiometric calibration and threshold segmentation, to extract spectral reflectance values for each experimental plot. Second, variance analysis and significance tests were conducted on three spring wheat varieties. Combining Pearson correlation and feature importance, vegetation indices were dynamically screened to construct an NNI estimation model using Bayesian optimized random forests. Finally, the influence patterns of NNI under different planting densities and nitrogen application rates were analyzed, and the optimal NNI estimation model was determined.

### 2.2 Overview of the study area and experimental design

**Figure 2:**
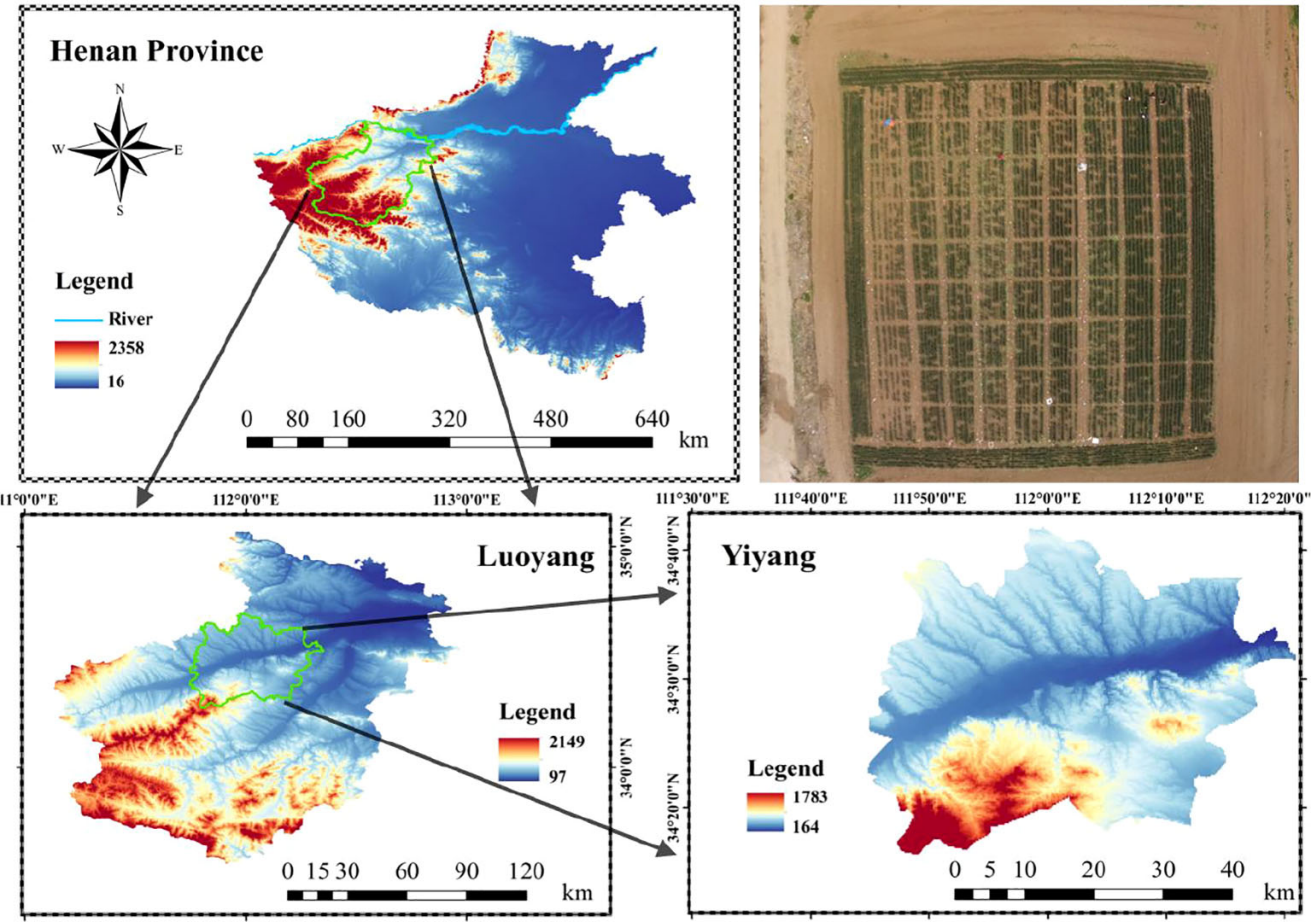
Geographic location of the study area.

The study area located in the Yiyang Digital Agriculture Industry Park, Lianzhuang Town, Yiyang County, Luoyang City, Henan Province (111°97′11.72″E, 34°47′79.03″N). The region features a temperate monsoon climate, with an average annual temperature of about 15 °C, annual precipitation ranging from 500 to 800 mm, a frost-free period of approximately 200 days, and annual sunshine duration between 1847.1 and 2313.6 hours. The geographical location of the study area is shown in **Figure 2**.

**Figure 3:**
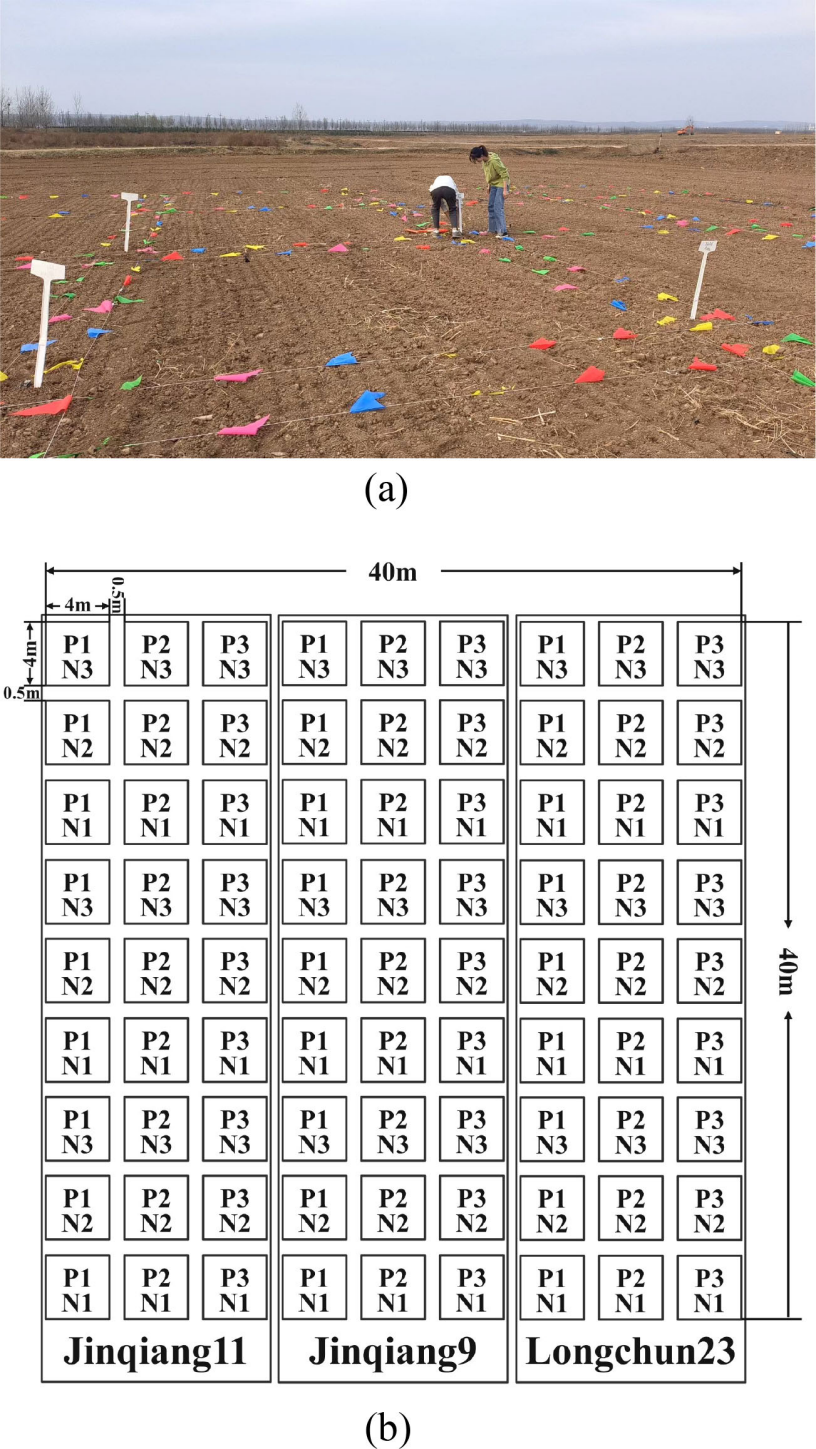
Setting and distribution of experimental plot areas. **(a)** Photos of the experimental area setup, **(b)** Distribution of the experimental area.

The experiment selected three spring wheat cultivars(Jinqiang11, Jinqiang9, and Longchun23), which are known for their strong resistance to lodging, high quality, and high yield. The seeds were sown on March 7, 2023, and harvested on July 3, 2023. Three nitrogen application levels were established, with urea applied as the base fertilizer at rates of 150 kg/hm^2^, 210 kg/hm^2^, and 270 kg/hm^2^, labeled as N1, N2, and N3, respectively. Three planting densities were set: 1.0 million plants/hm^2^, 1.6 million plants/hm^2^, and 2.2 million plants/hm^2^, marked as P1, P2, and P3, respectively. The experiment followed a complete factorial design with plot planting, and each treatment was replicated three times, resulting in a total of 81 plots. The layout of the experimental plots is shown in **Figure 3**.

Wheat was sown using a seed drill and irrigated via sprinkling. Other management practices adhered to local standards for wheat field management. Prior to the experiment, in order to understand the soil nutrient status and to obtain an unbiased estimate of the initial state of the whole experimental plot, and to provide reliable baseline data for subsequent experiments (Jing et al., 2025), three groups of soil samples were randomly taken from the experimental area for nutrient analysis., with the results presented in **Table 1**.

**Table 1:** Soil nutrient analysis.

Soil nutrient content	Sample 1	Sample 3	Sample 3	Average
Total nitrogen g/kg	0.137	0.088	0.109	0.111
Total phosphorus g/kg	7.927	6.680	8.930	7.846
Total potassium g/kg	8.574	7.579	8.263	8.139
Alkaline hydrolyzable nitrogen g/kg	40.973	58.876	48.147	49.332
Available phosphorus g/kg	256.969	291.267	292.687	280.307
Available potassium g/kg	204.474	163.457	176.17	181.367
Organic carbon g/kg	2.881	7.465	8.092	6.146

### 2.3 Data collection and processing

**Figure 4:**
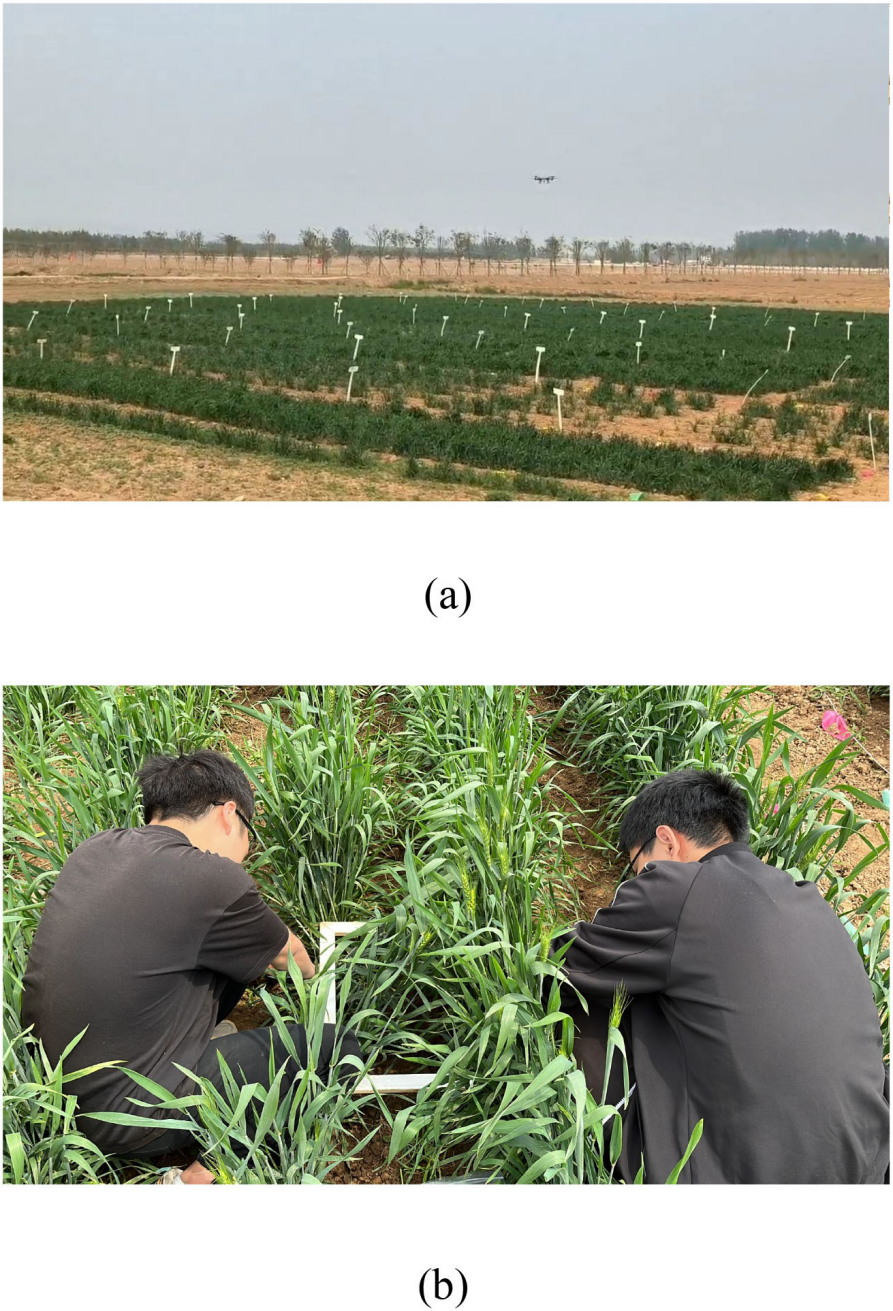
Data acquisition process. **(a)** UAV multispectral data acquisition, **(b)** Field measurements Collection.

UAV multispectral data were collected on April 12, April 26, May 8, and May 24, corresponding to the four growth stages of spring wheat (tillering, jointing, booting, and grain filling). Field measurements were conducted simultaneously with the multispectral data collection (**Figure 4**).

A DJI Phantom 4 Multispectral (P4M) UAV (DJI, Shenzhen, China) was used to acquire multispectral remote sensing images of the study area. This device includes one color sensor for visible light imaging and five monochrome sensors for multispectral imaging: blue (B) at 450 nm, green (G) at 560 nm, red (R) at 650 nm, red-edge (RE) at 730 nm, and near-infrared (NIR) at 840 nm. Data were collected on sunny, cloudless days with wind less than 3 Beaufort, between 10:00 a.m. and 3:00 p.m. The flight altitude was 30 m, with 80% forward overlap and 70% side overlap. Before the UAV took off, MAPIR standard calibration plates with reflectance of 25%, 50%, 75% and 95% were placed flat on the ground and images of the standard calibration plates were captured for radiometric correction.

In the data preprocessing stage, multiple incomplete images collected by the UAV were imported into DJI Zhitu for image stitching to generate a complete image of the study area, providing accurate basic data for subsequent remote sensing analysis and application. In order to eliminate the radiometric aberrations caused by light intensity, weather conditions and other factors, the standard radiometric version of the captured image was used to radiometrically correct the acquired multispectral image. The centimeter-level ground control point coordinates were determined using a GNSS receiver-G970II Pro high-precision RTKGNSS measurement system (UniStrong, Beijing, China) to provide accurate georeferencing. The coordinates of the determined ground control points were imported into ENVI5.6, and according to the positions and coordinates of the control points, the projection and distortion of the images were adjusted to be consistent with the actual situation on the ground, and orthophotos of the study area were obtained. The image was cropped using Matlab R2020a, and after removing the non-study area, the cropped orthophoto was processed using the Enhanced Vegetation Index EVI to obtain a binarized grayscale mask image, which facilitates the segmentation of the background information from the wheat plants; the influence of the wheat plants in each test plot was extracted using Matlab R2020a as the region of interest for that test plot (ROI), and take the average value of reflectance of all pixel points in the ROI range, which is the reflectance spectrum of the plot.

For field measurements, 0.5 m × 0.5 m areas within each plot were randomly selected to collect aboveground biomass samples. After removing dust, soil, and other debris, the samples were placed in a forced-air oven at 105 °C for 30 minutes to kill the green tissue, followed by drying at 80 °C for 24–72 hours until constant weight was achieved. This dry weight was recorded and converted to above ground biomass (AGB) per hectare (t/hm²) based on the total area of the sampling points. The dried samples were ground and analyzed for plant nitrogen concentration (PNC) using the Kjeldahl method. The nitrogen nutrition index (NNI) was calculated as the ratio of plant nitrogen concentration to critical nitrogen concentration, using the following formulas:

(1) $$NNI=N_{c}/N_{ct}$$

(2) $$N_{ct}=4.15W^{-0.38}$$

Where, $N_{c}$ denotes the plant nitrogen concentration (%), $N_{ct}$ denotes the critical nitrogen concentration (%), and W denotes the above ground biomass (t/hm^2^). The critical nitrogen dilution curve model for wheat in the North China Plain established by Wang (Wang et al., 2012) is employed in this study (Equation 2).

A total of 324 samples were collected in this study, with 315 remaining after manually removing damaged samples.

### 2.4 Calculation of vegetation indices

Changes in nitrogen levels can modify the chlorophyll content (Linders et al., 2024) and cellular structure (Macadam et al., 1989) of plant leaves, thereby influencing the absorption and reflection characteristics in the visible and near-infrared regions. Vegetation indices are dimensionless parameters constructed through linear or nonlinear combinations of reflectance from different spectral bands in remote sensing images, based on the unique spectral absorption characteristics of vegetation. These indices effectively characterize the reflectance features of vegetation in the visible and near-infrared regions and their differences from the soil background, serving as a concise and practical empirical indicator for quantifying surface vegetation status. In this study, 23 vegetation indices reported in the literature were selected, with their formulas presented in **Table 2**.

**Table 2:** Vegetation indices selected.

Vegetation indices	Calculation equation	Reference
GNDVI	$\text{GNDVI}={\text{(R}}_{840}-{\text{R}}_{560})/{\text{(R}}_{840}+{\text{R}}_{560})$	(Jiang et al., 2022)
MNVI	$\text{MNVI}=\text{(1}{.5\times R}_{840}^{2}-1.5\times {\text{R}}_{650})/{\text{(R}}_{840}^{2}+{\text{R}}_{650}+0.5)$	(Qi, 2023)
NDVI	$\text{NDVI}={\text{(R}}_{840}-{\text{R}}_{650})/{\text{(R}}_{840}+{\text{R}}_{650})$	(Choudhury et al., 2021)
OSAVI	${\text{OSAVI=(R}}_{840}{\text{-R}}_{650})/{\text{(R}}_{840}{\text{+R}}_{650}+0 .16)$	
EVI	$\text{EVI}=2.5\times {\text{(R}}_{840}-{\text{R}}_{650})/(1+{\text{R}}_{840}+6\times {\text{R}}_{650}-7.5\times {\text{R}}_{450})$	
RVI	$\text{RVI}={\text{R}}_{840}/{\text{R}}_{650}$	(Anchal et al., 2022)
CIVE	$\text{CIVE}=0.441\times {\text{R}}_{650}-0.881\times {\text{R}}_{560}+0.385{\text{×R}}_{650}+18.78745$	
ExG	$\text{E}\times \text{G}=2\times {\text{R}}_{560}-{\text{R}}_{650}-{\text{R}}_{450}$	
VARI	$\text{VARI}={\text{R}}_{560}-{\text{R}}_{650}/{\text{R}}_{560}+{\text{R}}_{650}-{\text{R}}_{450}$	
ENDVI	$\text{ENDVI}={\text{(R}}_{840}+{\text{R}}_{560}-2\times {\text{R}}_{450})/{\text{(R}}_{840}+{\text{R}}_{560}+2\times {\text{R}}_{450})$	
SAVI	$\text{SAVI}=(1+0.5)\times {\text{(R}}_{840}-{\text{R}}_{650})/{\text{(R}}_{840}+{\text{R}}_{650}+0.5)$	(Han et al., 2022a)
RTVICore	${\text{RTVI}}_{Core}=100\times {\text{(R}}_{840}-{\text{R}}_{730})-10\times {\text{(R}}_{840}-{\text{R}}_{560})$	
SRREDEDGE	$\text{SRREDEDGE}={\text{R}}_{840}/{\text{R}}_{730}$	
EVI2	$\text{EVI}2=2.4\times {\text{(R}}_{840}-{\text{R}}_{650})/{\text{(R}}_{840}+{\text{R}}_{650}+1)$	
MCARI	$\text{MCARI}={\text{R}}_{730}\times {\text{((R}}_{730}-{\text{R}}_{650})-(0.2\times {\text{(R}}_{730}-{\text{R}}_{560}\text{)))}/{\text{R}}_{650}$	
DVI	$\text{DVI}={\text{R}}_{840}-{\text{R}}_{650}$	
GRVI	$\text{GRVI}={\text{R}}_{840}/{\text{R}}_{560}$	(Han et al., 2022b)
NGI	$\text{NGI}={\text{R}}_{560}/{\text{(R}}_{840}+{\text{R}}_{560}+{\text{R}}_{730})$	
NREI	$\text{NREI}={\text{R}}_{730}/{\text{(R}}_{840}+{\text{R}}_{560}+{\text{R}}_{730})$	
NNIR	$\text{NNIR}={\text{R}}_{840}/{\text{(R}}_{840}+{\text{R}}_{650}+{\text{R}}_{730})$	
MDD	$\text{MDD}=({\text{R}}_{840}-{\text{R}}_{730})/{\text{(R}}_{730}-{\text{R}}_{560})$	
MNDI	$\text{MNDI}={\text{(R}}_{840}-{\text{R}}_{730})/{\text{(R}}_{840}-{\text{R}}_{560})$	
MEVI	$MEVI=2.5\times \left(R_{840}-R_{730}\right)/\left(R_{840}+6\times R_{730}-7.5\times R_{560}+1\right)$	

In the table, ${\text{R}}_{450}$, ${\text{R}}_{560}$, ${\text{R}}_{650}$, ${\text{R}}_{730}$ and ${\text{R}}_{840}$ denote the spectral reflectance of the blue, green, red, red-edge, and near-infrared bands, respectively.

### 2.5 Data analysis and modeling

#### 2.5.1 Feature variable selection

(1)Pearson correlation coefficient.

The Pearson correlation coefficient quantifies the strength of the linear relationship between two variables (Van Den Heuvel and Zhan, 2022). The closer its absolute value is to 1, the stronger the relationship between the variables. Specifically, when |r|≤0.8, the two variables are highly correlated; when 0.5≤|r|<0.8, they are moderately correlated; when 0.3≤|r|<0.5, they are weakly correlated; and when |r|<0.3, the two variables are considered to be essentially uncorrelated.

(2)Feature importance.

Random Forest-based feature importance analysis can measure the degree of contribution of features to improve the accuracy of model prediction, capturing the nonlinear relationship and interaction effect between features and target variables (Chen et al., 2023), the higher the score of feature importance, the more critical the feature is to make accurate predictions in the model.

In order to comprehensively assess the contribution of features to the prediction task and to screen for more discriminative features, this study employs both of the above methods with the aim of balancing linear and nonlinear information in the screening process. To optimize model inputs and improve model accuracy and efficiency, this study calculated the Pearson correlation coefficients and relative feature importance between the 23 vegetation indices and NNI under different treatments. The results were then sorted, and vegetation indices with higher absolute correlation coefficients and feature importance were gradually retained as model inputs. In this study, the number of retained vegetation indices was sequentially 23, 20, 15, 10, 5, 3, and 1.

#### 2.5.2 Modeling methods

In this study, Bayesian Optimization (BO) (Sun et al., 2021) Random Forest (RF) models were developed using varying numbers of vegetation indices as inputs. RF is a machine learning method based on ensemble learning (Schonlau and Zou, 2020). Its core principle involves constructing multiple independently trained decision trees and integrating their prediction results to enhance model performance. Owing to its high prediction accuracy, strong robustness, and broad applicability, RF has become a widely used and efficient algorithm in the field of machine learning. The performance of machine learning models is highly dependent on hyperparameters, making the selection of optimal parameters using effective algorithms crucial. Traditional methods, such as grid search, are limited due to low computational efficiency. In this study, the BO algorithm was employed to minimize the loss function of the machine learning model. By constructing a prior function through Gaussian process regression and combining acquisition functions such as Expected Improvement (EI), Probability of Improvement (PI), and Upper Confidence Bound (UCB), the optimal combination of hyperparameters was identified through steps including random sampling, surrogate function establishment, posterior distribution update, and iterative optimization, thereby achieving hyperparameter tuning. In this study, Bayesian optimization is used to find the optimality of three core hyperparameters of random forests, which are the number of decision trees (minTree), the minimum number of samples of leaf nodes (minLS), and the number of features considered in each segmentation (numPTS). minTree’s range of variation is set to be from 5 to 200. minLS’s range of variation is from 1 to 20. numPTS’s range is from 1 to n-1, and n is the number of training samples.

In this study, 23 vegetation indices were used as independent variables, and the wheat nitrogen nutrition index (NNI) was used as the dependent variable to construct NNI estimation models for the entire growth period, each of the four individual growth stages, each of the three nitrogen levels, each of the three planting densities, and each of the three cultivars, based on RF and BO-RF. Due to the limitation of sample size, the leave-one-out cross-validation method was employed. Each training session used only one data point as the test set and the remaining data as the training set (Liu et al., 2020).It has been shown that the method is more suitable for small sample datasets (Zhu et al., 2022), which allows the training results to be closest to the expectations of the entire test set.

#### 2.5.3 Model evaluation methods

This study utilized three metrics to assess model accuracy: the coefficient of determination (R^2^), root mean square error (RMSE), and mean absolute error (MAE). R^2^ indicates the degree of fit between predicted and measured values (Nyasulu et al., 2022). RMSE reflects the deviation between predicted and measured values. A larger R^2^ and a smaller RMSE signify higher model accuracy and better performance. MAE reflects the difference between predicted and actual values, with smaller MAE values indicating higher model prediction accuracy.

#### 2.5.4 Data processing and analysis

Data processing was performed using WPS Office software. Pearson correlation analysis, one-way ANOVA, variance analysis, and significance tests were conducted using IBM SPSS Statistics 27. Feature importance calculation was implemented using VS Code and Python 3.9. The BO-RF model was constructed using MATLAB R2023b.

## 3 Results

### 3.1 Distribution of NNI under different treatments

**Figure 5:**
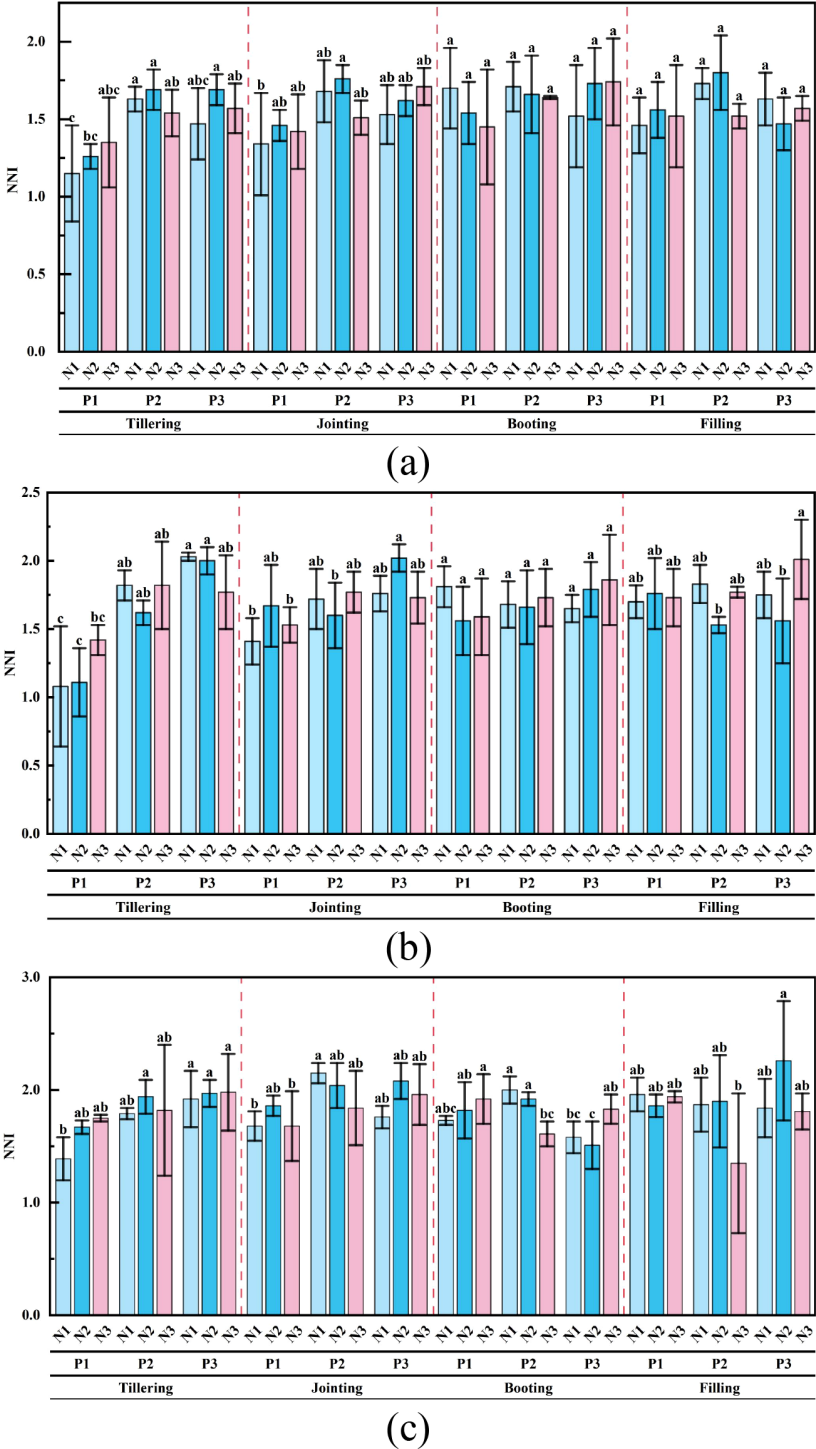
Characteristics of NNI changes under the coupled effects of planting density and nitrogen application rate for the three spring wheat cultivars. **(a)** Jinqiang11, **(b)** Jinqiang9, **(c)** Longchun23. Different lowercase letters within the same growth stage indicate significant differences at the 0.05 level.

**Figure 5** shows the changes in NNI for three spring wheat varieties under coupled planting density and nitrogen application levels.

It can be observed that the NNI variation trends of different spring wheat cultivars across various growth stages are generally similar. Overall, the NNI in most treatments tends to increase with the growth stage, reaching a relatively high value at the booting stage and showing slight fluctuations during the grain-filling stage.

Significant differences in NNI were found among different treatments for each cultivar. Under the same planting density, the NNI did not show a regular change trend with increasing nitrogen application rate. For Jinqiang11, there were no significant differences in NNI among different nitrogen levels. For Jinqiang9, at the P3 planting density during the grain-filling stage, the NNI under the N3 treatment was significantly higher than that under the N2 treatment, with an increase of 28.85%. For Longchun23, at the P2 planting density during the booting stage, the NNI under both the N1 and N2 treatments was significantly higher than that under the N3 treatment, with increases of 24.22% and 19.25%, respectively; at the P3 planting density, the NNI under the N3 treatment was significantly higher than that under the N2 treatment, with an increase of 21.19%.

Under the same level of N application, with the increase of P, the overall NNI showed a trend of “rising first and then leveling off”, which was P3 ≈ P2 > P1. It is worth noting that the NNI of P2 was slightly larger than that of P3 at certain fertility stages, such as the tillering, jointing, and filling stages of Jinqiang 11. For Jinqiang11, at the tillering stage, under the N1 nitrogen level, the NNI under the P2 treatment was significantly higher than that under the P1 treatment, with an increase of 41.74%; under the N2 nitrogen level, the NNI under both the P2 and P3 treatments was significantly higher than that under the P1 treatment, with an increase of 34.13%. For Jinqiang9, at the tillering stage, under the N1 nitrogen level, the NNI under both the P2 and P3 treatments was significantly higher than that under the P1 treatment, with increases of 68.52% and 87.96%, respectively; under the N2 nitrogen level, the NNI under both the P2 and P3 treatments was significantly higher than that under the P1 treatment, with increases of 45.95% and 80.18%, respectively; at the jointing stage, under the N2 nitrogen level, the NNI under the P3 treatment was significantly higher than that under the P2 treatment, with an increase of 26.25%. For Longchun23, at the tillering stage, under the N1 nitrogen level, the NNI under the P3 treatment was significantly higher than that under the P1 treatment, with an increase of 38.13%; at the jointing stage, under the N1 nitrogen level, the NNI under the P2 treatment was significantly higher than that under the P1 treatment, with an increase of 28.98%; at the booting stage, under the N1 nitrogen level, the NNI under the P2 treatment was significantly higher than that under the P3 treatment, with an increase of 26.58%; under the N2 nitrogen level, the NNI under the P2 treatment was significantly higher than that under the P3 treatment, with an increase of 27.15%; under the N3 nitrogen level, the NNI under the P1 treatment was significantly higher than that under the P2 treatment, with an increase of 19.25%.

Significance tests (*F*-values) were performed on the three spring wheat cultivars under the coupled effects of planting density and nitrogen application rate, with the results presented in **Table 3**.

**Table 3:** Analysis of variance for the coupled effects of planting density and nitrogen application rate on NNI in spring wheat.

Varieties	Source of variation	Tillering	Jointing	Booting	Filling
Jinqiang11	P	17.823 ***	2.573 ns	0.358 ns	2.980 ns
	N	0.850 ns	0.815 ns	0.052 ns	0.296 ns
	P×N	0.592 ns	1.179 ns	0.655 ns	1.034 ns
Jinqiang9	P	26.780 ***	17.628 ***	0.910 ns	0.157 ns
	N	0.359 ns	5.010 *	0.349 ns	3.256 ns
	P×N	1.736 ns	0.829 ns	0.319 ns	1.563 ns
Longchun23	P	5.357 *	5.544 *	1.574 ns	0.988 ns
	N	28.862 ***	2.375 ns	0.476 ns	3.593 ns
	P×N	0.319 ns	1.112 ns	6.844 **	1.499 ns

*, indicates a significant difference (*p* < 0.1); **, indicates a more significant difference (*p* < 0.05); ***, indicates an extremely significant difference (*p* < 0.01); and ns indicates non-significance.

The results of the significance tests show that the effects of planting density, nitrogen application rate, and their interaction on NNI in spring wheat vary among different cultivars and growth stages.

For Jinqiang11, planting density had an extremely significant difference effect on NNI at the tillering stage (*p* < 0.01), but no significant effect at other growth stages; nitrogen application rate had no significant effect at any stage, and the interaction between planting density and nitrogen application rate was not significant at any stage. For Jinqiang9, planting density had an extremely significant difference effect on NNI at the tillering and jointing stages (*p* < 0.01), and nitrogen application rate had a more significant difference effect at the jointing stage (*p* < 0.05), however, the interaction was not significant at any stage. For Longchun23, planting density had a more significant difference effect on NNI at the tillering and jointing stages (*p* < 0.05), nitrogen application rate had an extremely significant difference effect at the tillering stage (*p* < 0.01), and the interaction was significant at the booting stage (*p* < 0.1). This indicates that the effects of planting density and nitrogen application rate on NNI vary by cultivar and growth stage. Overall, the coupled effect of planting density and nitrogen application rate on NNI in spring wheat is relatively small.

### 3.2 Analysis of the relative importance of vegetation indices for NNI

**Figure 6:**
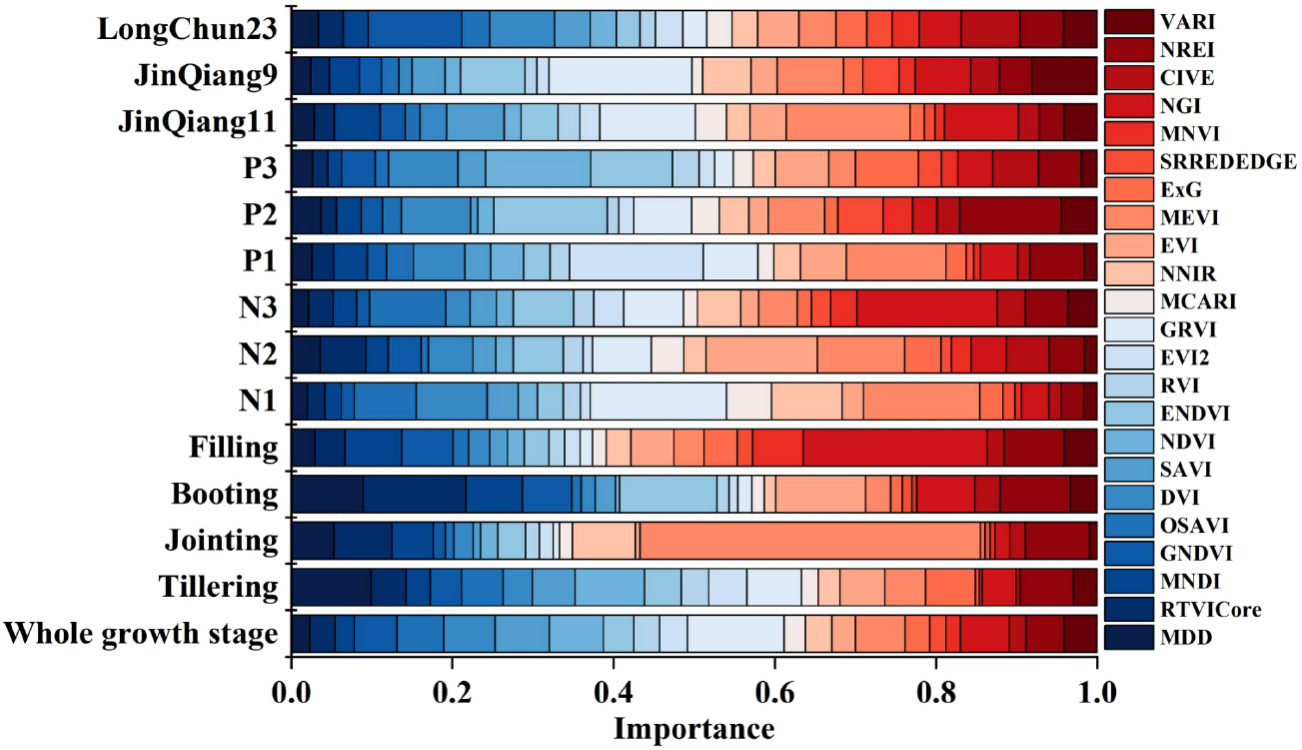
Importance of vegetation indices for NNI under different conditions. Different colors represent different vegetation indices, and the length of the color block represents the magnitude of feature importance.

This study used RF to calculate the relative importance of 23 vegetation indices for NNI under different treatments, providing a reference for feature selection. The results are shown in **Figure 6**.

The importance of different vegetation indices varied significantly under different treatments. Among the four individual growth stages, the importance of the best vegetation index for NNI ranged from 0.099 to 0.422. The best vegetation index during the jointing stage, MEVI, had the highest importance (0.422), while the best vegetation index during the tillering stage, MDD, had the lowest importance (0.099). The best vegetation indices during the booting and grain-filling stages were RTVICore and NGI, respectively, with importance of 0.128 and 0.228. Across the entire growth period, GRVI had the highest importance (0.120). Under different nitrogen levels, the importance of the best vegetation index for NNI ranged from 0.138 to 0.174. The best vegetation indices under the N1, N2, and N3 treatments were GRVI (0.169), EVI (0.138), and NGI (0.174), respectively. Under different planting densities, the importance of the best vegetation index for NNI ranged from 0.130 to 0.166. Under low planting density (P1), EVI2 and MEVI had relatively high importance values of 0.166 and 0.123, respectively. The best vegetation indices under the P2 and P3 treatments were ENDVI and NDVI, with importance of 0.141 and 0.130, respectively. Among the three spring wheat cultivars, the importance of the best vegetation index for NNI ranged from 0.117 to 0.177. For Jinqiang9, GRVI had the highest importance (0.177). For Jinqiang11 and Longchun23, the best vegetation indices were MEVI and GNDVI, with importance of 0.154 and 0.117, respectively.

### 3.3 Correlation analysis between vegetation indices and NNI

**Figure 7:**
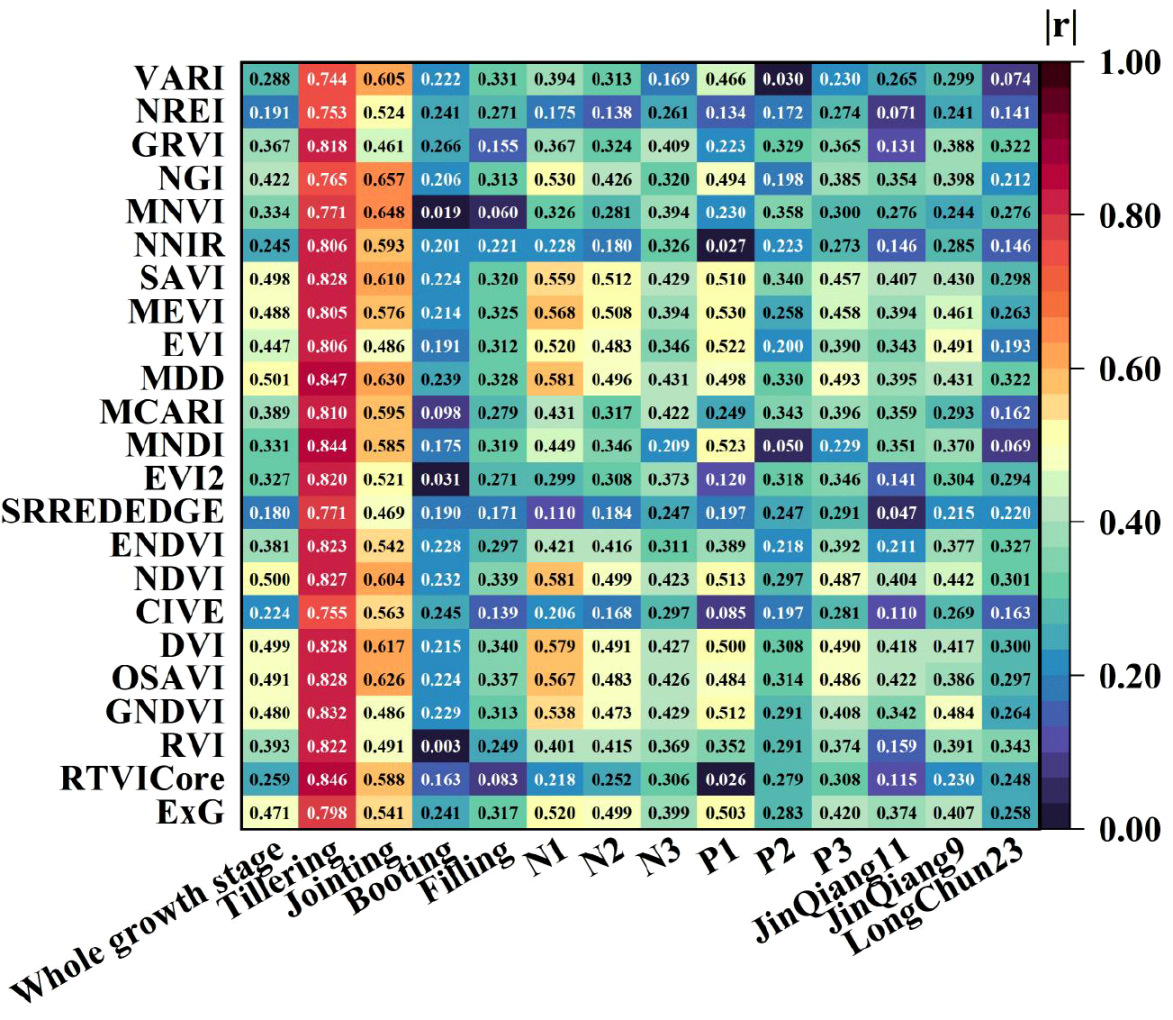
Correlation between vegetation indices and NNI under different treatments.

This study calculated the Pearson correlation between the 23 vegetation indices and NNI under different treatments. The absolute values of the correlation coefficients are shown in **Figure 7**, and the significance results are presented in **Table 4**.

**Table 4:** Significance of the correlation between vegetation indices and NNI under different treatments.

Vegetation index	Whole growth stage	Tillering	Jointing	Booting	Filling	N1	N2	N3	P1	P2	P3	Jinqiang 11	Jinqiang 9	Longchun 23
VARI	**	**	**	*	**	**	**	–	**	–	*	**	**	–
NREI	**	**	**	*	*	–	–	**	–	–	**	–	*	–
CIVE	**	**	**	*	–	*	–	**	–	*	**	–	**	–
NGI	**	**	**	–	**	**	**	**	**	*	**	**	**	*
MNVI	**	**	**	–	–	**	**	**	*	**	**	**	*	**
SRREDEDGE	**	**	**	–	–	–	–	*	*	*	**	–	*	*
ExG	**	**	**	*	**	**	**	**	**	**	**	**	**	**
MEVI	**	**	**	–	**	**	**	**	**	**	**	**	**	**
EVI	**	**	**	–	**	**	**	**	**	*	**	**	**	*
NNIR	**	**	**	–	–	*	–	**	–	*	**	–	**	–
MCARI	**	**	**	–	*	**	**	**	**	**	**	**	**	–
GRVI	**	**	**	*	–	**	**	**	*	**	**	–	**	**
EVI2	**	**	**	–	*	**	**	**	–	**	**	–	**	**
RVI	**	**	**	–	*	**	**	**	**	**	**	–	**	**
ENDVI	**	**	**	*	**	**	**	**	**	*	**	*	**	**
NDVI	**	**	**	*	**	**	**	**	**	**	**	**	**	**
SAVI	**	**	**	*	**	**	**	**	**	**	**	**	**	**
DVI	**	**	**	–	**	**	**	**	**	**	**	**	**	**
OSAVI	**	**	**	*	**	**	**	**	**	**	**	**	**	**
GNDVI	**	**	**	*	**	**	**	**	**	**	**	**	**	**
MNDI	**	**	**	–	**	**	**	*	**	–	*	**	**	–
RTVICore	**	**	**	–	–	*	**	**	–	**	**	–	*	*
MDD	**	**	**	*	**	**	**	**	**	**	**	**	**	**

**, indicates highly significant correlation at the 0.01 level (*p* < 0.01); *, indicates significant correlation at the 0.05 level (*p* < 0.05); and - indicates non-significant correlation.

The results indicate that for individual growth stages, all 23 vegetation indices in the tillering and jointing stages showed highly significant correlation with NNI. During the tillering stage, the correlation of vegetation indices was generally strong, with all correlation coefficients exceeding an absolute value of 0.740. Among them, MDD had the highest correlation coefficient of 0.847. In the jointing stage, NGI and MNVI exhibited relatively high correlation coefficients of 0.657 and 0.648, respectively. Except for RVI, EVI, GNDVI, SRREDEDGE, and GRVI, the correlation coefficients of the remaining vegetation indices were above 0.5, indicating moderate correlation. In the booting and grain-filling stages, the correlation between vegetation indices and NNI was generally low. The highest correlation coefficients were 0.266 for GRVI and 0.340 for DVI. Across the entire growth period, all 23 vegetation indices showed highly significant correlation with NNI, with MDD having the highest correlation coefficient of 0.501. The correlation between different vegetation indices and NNI varied significantly under different nitrogen application rates and planting densities. Under the N1 and N2 treatments, 10 and 2 vegetation indices, respectively, showed moderate correlation with NNI. Under the N3 treatment, the best vegetation index MDD had a correlation coefficient of only 0.431. The best vegetation indices under different planting densities were MEVI, MNVI, and MDD, with correlation coefficients of 0.530, 0.358, and 0.493, respectively, all showing significant correlation with NNI. The best vegetation indices for the three wheat cultivars were OSAVI, EVI, and RVI, with correlation coefficients of 0.422, 0.491, and 0.343, respectively, all showing significant correlation with NNI.

### 3.4 NNI estimation based on the BO-RF model

The relative importance of features and the Pearson correlation coefficients were calculated for different treatments. The vegetation indices were ranked and sequentially retained as 23, 20, 15, 10, 5, 3, and 1 vegetation index to establish the BO-RF model.

#### 3.4.1 NNI modeling analysis under different nitrogen application levels

**Table 5** shows the results of NNI estimation by the BO-RF models constructed under two vegetation index selection methods at three different nitrogen application levels.

**Table 5:** NNI modeling results under different nitrogen application levels.

Methods of feature screening	Number of vegetation indices	N1			N2			N3		
		R^2^	RMSE	MAE	R^2^	RMSE	MAE	R^2^	RMSE	MAE
Correlation	23	0.666	0.164	0.125	0.647	0.172	0.128	0.524	0.191	0.137
	20	0.683	0.161	0.123	0.640	0.174	0.129	0.528	0.190	0.136
	15	0.688	0.159	0.122	0.661	0.168	0.125	0.510	0.193	0.138
	10	0.692	0.157	0.121	0.698	0.159	0.116	0.465	0.200	0.144
	5	0.606	0.175	0.133	0.641	0.173	0.126	0.447	0.195	0.149
	3	0.467	0.203	0.155	0.544	0.193	0.142	0.436	0.205	0.153
	1	0.398	0.214	0.165	0.400	0.219	0.166	0.323	0.229	0.168
Importance	23	0.680	0.161	0.123	0.650	0.172	0.128	0.537	0.189	0.135
	20	0.663	0.165	0.126	0.670	0.167	0.124	0.551	0.186	0.132
	15	0.654	0.166	0.127	0.666	0.167	0.124	0.559	0.184	0.131
	10	0.673	0.162	0.124	0.754	0.145	0.106	0.541	0.188	0.133
	5	0.585	0.179	0.136	0.785	0.137	0.099	0.496	0.195	0.138
	3	0.466	0.203	0.154	0.560	0.188	0.143	0.499	0.195	0.139
	1	0.443	0.207	0.157	0.322	0.232	0.184	0.474	0.198	0.140

Under the N1 nitrogen application level, the models established with 10 and 23 vegetation indices based on correlation and feature importance, respectively, achieved the highest accuracy. Compared to the model using correlation, the model using feature importance had fewer vegetation indices and achieved better performance with R^2^ RMSE, and MAE values of 0.692, 0.157, and 0.121, respectively. Under the N2 and N3 nitrogen application levels, the models built with 5 and 15 vegetation indices selected based on feature importance performed the best, with R^2^, RMSE, and MAE values of 0.785 and 0.559, 0.137 and 0.184, 0.099 and 0.131, respectively. Compared to the models using correlation, the models using feature importance showed improved accuracy and reduced the number of vegetation indices by 50% and 25%, respectively. Among the three nitrogen levels, the models established under the N2 level using both feature selection methods achieved the highest accuracy, followed by N1, while the optimal model under the N3 level had the lowest accuracy (R^2^ = 0.559).

#### 3.4.2 NNI modeling analysis under different planting densities

**Table 6** shows the results of NNI estimation by the BO-RF models constructed under two vegetation index selection methods at three different planting densities.

**Table 6:** NNI modeling results under different planting densities.

Methods of feature screening	Number of vegetation indices	P1			P2			P3		
		R^2^	RMSE	MAE	R^2^	RMSE	MAE	R^2^	RMSE	MAE
Correlation	23	0.683	0.168	0.129	0.310	0.207	0.155	0.466	0.189	0.140
	20	0.704	0.162	0.124	0.293	0.209	0.156	0.460	0.190	0.141
	15	0.706	0.162	0.125	0.271	0.211	0.158	0.460	0.189	0.140
	10	0.699	0.163	0.127	0.272	0.210	0.155	0.478	0.186	0.138
	5	0.673	0.170	0.130	0.264	0.212	0.159	0.422	0.194	0.145
	3	0.649	0.177	0.134	0.206	0.218	0.159	0.497	0.182	0.134
	1	0.444	0.219	0.165	0.151	0.225	0.167	0.369	0.205	0.151
Importance	23	0.690	0.166	0.128	0.330	0.204	0.153	0.462	0.190	0.140
	20	0.687	0.166	0.128	0.322	0.205	0.154	0.475	0.187	0.139
	15	0.716	0.158	0.121	0.333	0.204	0.152	0.455	0.190	0.141
	10	0.681	0.168	0.129	0.311	0.207	0.155	0.467	0.187	0.139
	5	0.645	0.176	0.134	0.314	0.206	0.155	0.411	0.196	0.147
	3	0.610	0.184	0.140	0.304	0.206	0.156	0.423	0.194	0.148
	1	0.573	0.191	0.145	0.191	0.219	0.161	0.379	0.201	0.153

Under the P1 planting density, the optimal models established based on both feature selection methods used 15 vegetation indices as inputs. The model based on feature importance achieved R², RMSE, and MAE values of 0.716, 0.158, and 0.121, respectively, which were better than the model based on correlation (R^2^ = 0.706, RMSE = 0.162, MAE = 0.125). Under the P2 planting density, the optimal model used 15 vegetation indices selected based on feature importance, but the model accuracy was relatively poor, with an R^2^ value of only 0.333. Under the P3 planting density, the model with the highest accuracy was established using 3 vegetation indices selected based on correlation, with R^2^, RMSE, and MAE values of 0.497, 0.182, and 0.134, respectively, which were better than the model using 20 vegetation indices selected based on feature importance. Among the three planting densities, the optimal model under P1 had the highest accuracy, while the models under P2 and P3 had lower accuracy, with R^2^ values less than 0.5.

#### 3.4.3 NNI modeling analysis under different growth stages

**Table 7** shows the results of NNI estimation by the BO-RF models constructed under two vegetation index selection methods during the tillering, jointing, booting, grain-filling stages, and across the entire growth period.

**Table 7:** NNI modeling results under different growth stages.

Methods of feature screening	Number of vegetation indices	Whole growth stage			Tillering			Jointing			Booting			Filling		
		R^2^	RMSE	MAE	R^2^	RMSE	MAE	R^2^	RMSE	MAE	R^2^	RMSE	MAE	R^2^	RMSE	MAE
Correlation	23	0.575	0.184	0.136	0.789	0.154	0.115	0.599	0.165	0.126	0.176	0.204	0.159	0.262	0.234	0.159
	20	0.586	0.182	0.134	0.788	0.154	0.115	0.597	0.165	0.126	0.186	0.203	0.158	0.262	0.234	0.158
	15	0.585	0.182	0.134	0.783	0.156	0.116	0.626	0.159	0.121	0.168	0.205	0.160	0.384	0.215	0.145
	10	0.573	0.184	0.136	0.781	0.156	0.116	0.670	0.150	0.115	0.176	0.204	0.159	0.249	0.237	0.161
	5	0.535	0.191	0.141	0.777	0.158	0.117	0.471	0.190	0.149	0.157	0.206	0.162	0.322	0.226	0.154
	3	0.513	0.195	0.145	0.761	0.163	0.121	0.412	0.200	0.155	0.160	0.206	0.160	0.171	0.247	0.167
	1	0.185	0.251	0.186	0.761	0.763	0.122	0.205	0.232	0.181	0.139	0.208	0.162	0.161	0.249	0.169
Importance	23	0.579	0.183	0.135	0.788	0.154	0.115	0.593	0.165	0.127	0.199	0.201	0.157	0.237	0.239	0.162
	20	0.573	0.184	0.136	0.789	0.154	0.115	0.604	0.163	0.125	0.182	0.204	0.159	0.264	0.234	0.159
	15	0.573	0.184	0.137	0.790	0.153	0.114	0.587	0.167	0.128	0.213	0.201	0.157	0.310	0.230	0.156
	10	0.557	0.187	0.139	0.798	0.150	0.111	0.594	0.166	0.126	0.269	0.194	0.153	0.287	0.235	0.161
	5	0.505	0.196	0.143	0.798	0.150	0.111	0.669	0.149	0.113	0.319	0.188	0.149	0.241	0.240	0.163
	3	0.491	0.199	0.147	0.784	0.155	0.116	0.579	0.168	0.131	0.632	0.143	0.112	0.153	0.250	0.173
	1	0.358	0.222	0.165	0.759	0.164	0.122	0.556	0.173	0.134	0.193	0.201	0.161	0.087	0.259	0.183

During the tillering and booting stages, the models established using 5 and 3 vegetation indices selected based on feature importance achieved the highest accuracy, with R^2^ values between 0.632 and 0.798, RMSE values between 0.143 and 0.150, and MAE values between 0.111 and 0.113. For the jointing and grain-filling stages, the optimal models used 10 and 15 vegetation indices selected based on correlation, with R^2^, RMSE, and MAE values of 0.670 and 0.384, 0.150 and 0.215, and 0.115 and 0.145, respectively. Among the four individual growth stages, the model constructed during the tillering stage had the highest accuracy (R^2^ = 0.798) and used fewer vegetation indices, while the model constructed during the grain-filling stage had the lowest accuracy (R^2^ = 0.384) and used the most vegetation indices. Across the entire growth period, the model with the highest accuracy used 20 vegetation indices selected based on correlation, with R^2^ = 0.586, RMSE = 0.182, and MAE = 0.134.

#### 3.4.4 NNI modeling analysis for different cultivars

**Table 8** shows the results of NNI estimation by the BO-RF models constructed under two vegetation index selection methods for three different spring wheat cultivars.

**Table 8:** NNI modeling results for different cultivars.

Methods of feature screening	Number of vegetation indices	Jinqiang11			Jinqiang9			Longchun23		
		R^2^	RMSE	MAE	R^2^	RMSE	MAE	R^2^	RMSE	MAE
Correlation	23	0.583	0.151	0.116	0.601	0.171	0.122	0.401	0.218	0.158
	20	0.589	0.150	0.115	0.602	0.170	0.122	0.408	0.216	0.155
	15	0.627	0.144	0.111	0.573	0.176	0.127	0.406	0.217	0.156
	10	0.599	0.148	0.115	0.619	0.166	0.119	0.392	0.218	0.156
	5	0.575	0.150	0.113	0.552	0.181	0.132	0.328	0.228	0.165
	3	0.362	0.178	0.134	0.488	0.191	0.141	0.283	0.236	0.170
	1	0.259	0.191	0.147	0.409	0.204	0.157	0.193	0.248	0.183
Importance	23	0.581	0.152	0.116	0.598	0.171	0.122	0.393	0.219	0.159
	20	0.580	0.152	0.116	0.596	0.172	0.123	0.383	0.221	0.160
	15	0.568	0.154	0.117	0.582	0.175	0.126	0.403	0.217	0.157
	10	0.563	0.153	0.117	0.581	0.175	0.123	0.354	0.225	0.165
	5	0.536	0.157	0.120	0.523	0.185	0.130	0.321	0.229	0.167
	3	0.510	0.160	0.121	0.499	0.188	0.133	0.375	0.220	0.161
	1	0.283	0.188	0.141	0.428	0.199	0.147	0.245	0.240	0.173

The optimal models for the three spring wheat cultivars were constructed using 15, 10, and 20 vegetation indices selected based on correlation, respectively. The R^2^ values ranged from 0.408 to 0.627, RMSE values from 0.144 to 0.216, and MAE values from 0.111 to 0.155. Among them, the estimation models for Jinqiang11 and Jinqiang9 had similar accuracy, with R^2^ values both above 0.6. The NNI estimation model for Longchun23 had the lowest accuracy, with R^2^ = 0.408, RMSE = 0.216, and MAE = 0.155.

## 4 Discussion

In this study, the influence law of planting agronomy on NNI estimation was explored, and UAV multispectral remote sensing was utilized to achieve nitrogen nutrient index (NNI) estimation in spring wheat under complex farmland environments. Machine learning was applied to diagnose crop nutrient requirements, and artificial intelligence technology was integrated with agricultural production (Farajpour, 2025). It opens up a new way for precise crop diagnosis and optimal management, provides innovation for the deep application of AI in complex agricultural production, and provides data and methodological support for the subsequent development of more robust agricultural models.

### 4.1 Effects of planting density and nitrogen application rate on NNI

Planting density and nitrogen application rate (Tian et al., 2025) are two key factors affecting crop growth, development, and nitrogen nutrition status. Planting density determines the number and spatial distribution of plants per unit area, which in turn affects inter-plant competition and resource utilization efficiency. Although higher planting density can increase the photosynthetic efficiency of the population, it may also lead to increased competition among plants, which affects nitrogen uptake and distribution (Ma et al., 2022). The level of nitrogen application directly affects the supply of nitrogen in the soil, which in turn affects the nitrogen nutritional status of the plant. Moderate N application can promote plant growth and improve the efficiency of nitrogen utilization, but excessive N application not only causes waste of N fertilizer, but also may lead to environmental pollution problems. In this study, NNI of spring wheat varieties showed significant differences under different treatments (**Figure 5**). At the same planting density, NNI as a whole did not show a regular trend with increasing N. Instead, NNI as a whole mostly showed a trend of P3 ≈ P2 > P1 as P increased. This may be due to the fact that, when P increases, the aboveground biomass will increase significantly (Yang et al., 2022), and according to the NNI calculation formulas (Equations 1, 2), NNI will be elevated accordingly. While P2 was slightly larger than P3 in some fertility periods, it might be due to the fact that P had already exceeded the optimal planting density at that time, resulting in increased competition among plants and decreased individual nitrogen concentration (Schofield et al., 2019), which led to a slower or even a fall in the growth rate of NNI.

### 4.2 Comparative analysis with other researches

Liu et al (Liu et al., 2018). conducted a field experiment with six nitrogen application levels and two wheat varieties, collecting NNI and drone multispectral data at four critical growth stages of winter wheat (regreening stage, jointing stage, heading stage, and flowering stage). Using different vegetation indices as inputs, four vegetation index-based NNI estimation models were constructed for each critical growth stage. The optimal models had determination coefficients of 0.64, 0.78, 0.90, and 0.95, respectively. The R^2^ values for the joint study were higher than those in this study during the jointing stage (R^2^ = 0.670) and heading stage (R^2^ = 0.632), but the multi-factor interaction experimental design in this study better reflects the complexity of actual farmland conditions.

The sensitivity of vegetation indices varies among different crop types, possibly due to differences in leaf and canopy structure among crops. Xu et al (Xu et al., 2021). designed a rice field trial with two varieties and five nitrogen fertilizer gradients, measuring the reflectance spectra of leaves at different leaf positions and plant NNI during key growth stages. Different NNI monitoring models were constructed for leaves at various positions. The results showed that the accuracy of the models varied across leaf positions, with the top three leaves exhibiting the highest estimation accuracy R^2^ = 0.731, RMSE = 0.130, which was lower than the highest model accuracy in this study at the N2 level of nitrogen application (R^2^ = 0.785, RMSE = 0.137). Chrysanthemum morifolium Ramat was used as the study subject by Wu et al (Wu et al., 2024). Hyperspectral reflectance data and NNI were collected for three leaf layers during the critical fertility period, and dual-band spectral index DVI-L1 and PLSR estimation models were constructed. The results showed that the PLSR-L1 model with the first layer of leaf spectra as input was optimal, with R^2^ and RMSE of 0.8177 and 0.2000, respectively. The effect of leaf position on the estimation accuracy of NNI was not taken into account in this study, and this should be added in subsequent studies, and the spectral data were collected using a multispectral drone, and a narrower and more continuous waveband hyperspectral camera to mine new vegetation indices for higher precision estimation. However, in contrast, this study set up a more comprehensive 3-factor experiment, which better revealed the influence of P and N on the estimation of NNI, and has a higher feasibility and potential for popularization in practical production.

### 4.3 NNI modeling analysis under planting density and nitrogen application rate

This study calculated the relative importance and Pearson correlation coefficients of 23 vegetation indices with NNI, finding that the correlation coefficients and importance rankings of the vegetation indices varied under different treatments. In terms of relative importance, vegetation indices such as MEVI, NGI, and NREI consistently ranked among the top 15 across all treatments (**Figure 6**), indicating their strong universality and stability across different growth stages, nitrogen levels, planting densities, and cultivars. This may be because these three vegetation indices all incorporate the green, red-edge, and near-infrared bands. Some studies have shown that the green light region is located in the reflective peak of chlorophyll (Xu and Ye, 2023), and the light is scattered many times to obtain deeper canopy information, the red border region is highly sensitive to chlorophyll content (Bai et al., 2025) and can reflect crop nitrogen nutrient status, and the near-infrared region can reflect biomass and canopy structure (Li et al., 2020), which can comprehensively reflect information on chlorophyll content, nitrogen accumulation (Cao et al., 2013), biomass, canopy structure, and canopy health, thereby providing a more comprehensive assessment of the crop’s nitrogen nutrition status. In contrast, vegetation indices such as MCARI, MNVI, RVI, and SRREDEDGE had importance values below 0.050 in most treatments, indicating limited contributions to NNI prediction. It may be due to the fact that these vegetation indices usually rely only on the red, red-edge, and near-infrared bands, resulting in limited information content and poorer performance across different treatments. In terms of correlation, vegetation indices like NDVI, MDD, DVI, and SAVI showed high correlation with NNI in most treatments (**Figure 7**), suggesting strong linear relationships and suitability for nitrogen monitoring under various conditions. Vegetation indices such as NNIR, NREI, and SRREDEDGE exhibited low correlation in most conditions. The results from the two selection methods were not entirely consistent, likely due to the different principles of the methods: RF captures non-linear relationships and feature interactions, while Pearson coefficients only measure linear correlation. The relationship between vegetation indices and NNI may be non-linear or involve interactions. The simultaneous use of both methods allows for a more comprehensive assessment of vegetation indices and thus optimizes the inputs to the model.

The order of vegetation indices used in the optimal models under different treatments is shown in **Table 9**. It can be seen that vegetation indices such as DVI, MDD, NGI, MEVI, NDVI, EVI, and ENDVI were frequently used as inputs in the optimal models across 14 treatments, indicating their strong robustness and suitability for complex and variable field environments.

**Table 9:** Ranking of vegetation indices in the optimal models under different conditions.

Rank	Whole growth stage	Tillering	Jointing	Booting	Filling	N1	N2	N3	P1	P2	P3	JinQiang11	JinQiang9	LongChun23
1	MDD	MDD	NGI	RTVICore	DVI	NDVI	EVI	NGI	EVI2	ENDVI	MDD	OSAVI	EVI	RVI
2	NDVI	NDVI	MNVI	ENDVI	NDVI	MDD	MEVI	OSAVI	MEVI	NREI	DVI	DVI	GNDVI	ENDVI
3	DVI	GRVI	MDD	EVI	OSAVI	DVI	GRVI	ENDVI	GRVI	DVI	NDVI	SAVI	MEVI	GRVI
4	SAVI	NREI	OSAVI	–	VARI	MEVI	ENDVI	GRVI	NREI	GRVI	–	NDVI	NDVI	MDD
5	OSAVI	ExG	DVI	–	MDD	OSAVI	RTVICore	NNIR	DVI	MEVI	–	MDD	MDD	NDVI
6	MEVI	–	SAVI-	–	MEVI	SAVI	–	NREI	EVI	SRREDEDGE	–	MEVI	SAVI	DVI
7	GNDVI	–	VARI-	–	SAVI	GNDVI	–	MEVI	NGI	VARI	–	ExG	DVI	SAVI
8	ExG	–	NDVI-	–	MNDI	NGI	–	EVI2	MNDI	MDD	–	MCARI	ExG	OSAVI
9	EVI	–	MCARI-	–	ExG	EVI	–	VARI	NDVI	MNVI	–	NGI	NGI	EVI2
10	NGI	–	NNIR-	–	NGI	ExG	–	CIVE	OSAVI	NNIR	–	MNDI	RVI	MNVI
11	RVI	–	–	–	GNDVI	–	–	SAVI	ENDVI	MCARI	–	EVI	–	GNDVI
12	MCARI	–	–	–	EVI	–	–	MNVI	NNIR	MNDI	–	GNDVI	–	MEVI
13	ENDVI	–	–	–	ENDVI	–	–	RTVICore	SAVI	NGI	–	MNVI	–	ExG
14	GRVI	–	–	–	MCARI	–	–	DVI	RTVICore	CIVE	–	VARI	–	RTVICore
15	MNVI	–	–	–	EVI2	–	–	MNDI	MDD	GNDVI	–	ENDVI	–	SRREDEDGE
16	MNDI	–	–	–	–	–	–	–	–	–	–	–	–	NGI
17	EVI2	–	–	–	–	–	–	–	–	–	–	–	–	EVI
18	VARI	–	–	–	–	–	–	–	–	–	–	–	–	CIVE
19	RTVICore	–	–	–	–	–	–	–	–	–	–	–	–	MCARI
20	NNIR	–	–	–	–	–	–	–	–	–	–	–	–	NNIR
21	–	–	–	–	–	–	–	–	–	–	–	–	–	–
22	–	–	–	–	–	–	–	–	–	–	–	–	–	–
23	–	–	–	–	–	–	–	–	–	–	–	–	–	–

Among the NNI estimation models for the three nitrogen levels (**Table 5**), the optimal model under the high nitrogen (N3) level had the lowest accuracy (R^2^ = 0.559). This may be due to the reduced efficiency of nitrogen utilization in spring wheat under high nitrogen levels. After applying nitrogen above the demand threshold, some plant leaves grew in vain, nitrogen allocation was imbalanced (Xu et al., 2024), NNI no longer varied linearly with nitrogen application, and the vegetation index response situation became unstable, which weakened the correlation between NNI and NNI, and thus reduced the estimation ability of the model.

Among the NNI estimation models for the three planting densities (**Table 6**), the models under high planting densities (P2 and P3) had significantly lower accuracy than the P1 level. This may be due to intensified competition among plants under high-density planting (Zhang et al., 2023), resulting in restricted growth for some wheat plants due to insufficient light and water. Additionally, the canopy structure is more complex under high-density treatments. In contrast, low planting density (P1) provides a clear canopy structure with abundant growth resources, leading to consistent spring wheat growth and stable vegetation index responses.

When analyzing NNI modeling for different growth stages of spring wheat (**Table 7**), it was found that during the tillering stage, the model using five vegetation indices as inputs achieved the highest accuracy (R^2^ = 0.798). As the growth stage progressed, the accuracy of the optimal models gradually decreased, with the best model at the grain-filling stage having an R^2^ value of only 0.384 and using the most vegetation indices. This may be because the canopy structure is relatively simple during the tillering stage, with less background noise interference and more consistent spring wheat growth, resulting in lower data variability and easier model fitting. In contrast, during the grain-filling stage, the canopy structure is more complex, nitrogen is transferred from leaves to grains (Zhou et al., 2011), reducing the sensitivity of vegetation indices, and the inconsistent grain-filling rate can lead to significant growth differences among spring wheat plants, increasing data variability and making model fitting more challenging.

Among the NNI estimation models for the three cultivars (**Table 8**), the optimal models for Jinqiang11 and Jinqiang9 had similar accuracy, with R^2^ values above 0.6. However, the optimal model for Longchun23 had lower accuracy, with an R^2^ value of only 0.408 using 20 vegetation indices. This may be because Jinqiang series cultivars are strong-gluten wheat varieties that typically have a more erect plant type, fewer tillers (Liang et al., 2019; Wang et al., 2016), better canopy light transmittance, and clearer leaf distribution layers. These characteristics allow for more comprehensive spectral information acquisition, reflecting the overall plant status more accurately. In contrast, Longchun23, a medium-gluten wheat, has a relatively lower nitrogen demand and stronger tillering ability (Yuan and Yang, 2009), which can lead to severe canopy closure under high-density planting, making it difficult for UAV to penetrate the canopy and obtain accurate vegetation information, thereby reducing model accuracy.

### 4.4 Future work

The models in this study were developed based on three spring wheat cultivars: Jinqiang 11, Jinqiang 9, and Longchun 23. The ability of these models to estimate NNI for other cultivars needs further validation. Future research should aim to develop more universal NNI models for different cultivars. This study used a UAV-mounted multispectral camera to obtain canopy reflectance data throughout the spring wheat growth period. Future research could employ hyperspectral cameras to acquire richer spectral information and collect data over a longer time span to achieve higher-precision NNI estimation for spring wheat. Due to the limitation of data acquisition, this study is currently validated only for spring wheat in Henan Province, and has not been tested in other crops or regions. In the future, validation should be conducted in different regions to test the model’s ability to estimate NNI for other crops, and experiments with longer time spans should be carried out. The leave-one-out cross-validation method used in this study is suitable for the current small-sample scenario, but it may be that each training set is almost the same, which causes the model output to be extremely sensitive to a certain anomaly, making the estimation results not stable enough, and the computational load of this method is large, and the dataset can be expanded and the performance of methods such as k-fold cross-validation can be compared in future studies.

## 5 Conclusions

This study employed UAVs to capture multispectral imagery of wheat canopies under varying planting densities and nitrogen application rates. Vegetation indices were screened using Pearson correlation analysis and RF-based feature importance analysis. A Bayesian-optimized random forest model was established to estimate wheat NNI. The study analyzed the influence patterns of planting density and nitrogen application rates on wheat NNI across different growth stages. Key findings are as follows: As planting density increases, NNI generally follows an initial rise followed by a decline. However, with increasing nitrogen application rates, NNI does not exhibit a consistent overall trend. At the N2 nitrogen rate (210 kg/hm^2^), the NNI estimation model achieved the highest accuracy, with R^2^, RMSE, and MAE values of 0.785, 0.137, and 0.099, respectively. At the P1 planting density (1 million plants/hm²), the NNI estimation model achieved the highest accuracy, with R^2^, RMSE, and MAE values of 0.716, 0.158, and 0.121, respectively. Among the 23 selected vegetation indices, DVI, MDD, NGI, MEVI, NDVI, EVI, and ENDVI demonstrated strong resistance to interference. Models constructed using these indices exhibited high robustness. This study not only elucidate the influence patterns of planting density and nitrogen application rates on spring wheat NNl but also establish a nitrogen nutrition level model for wheat. This findings holds significant implications for assessing wheat growth at different growth stages and guiding the precise application of nitrogen fertilizers in agricultural fields.

## Data Availability

The raw data supporting the conclusions of this article will be made available by the authors, without undue reservation.
